# Controlling Selectivity of Surface Electro-Precipitation (SEP) in the Recovery of Rare Earth Elements (REE) from Aqueous Feedstocks

**DOI:** 10.1021/acssuschemeng.5c02403

**Published:** 2025-06-12

**Authors:** Irina V. Chernyshova, Wesam Tork, Sathish Ponnurangam

**Affiliations:** † Department of Earth and Environmental Engineering, Columbia University, New York, NY 10027, United States; ‡ Department of Chemical and Petroleum Engineering, 2129 University of Calgary, Calgary, Alberta T2N 1N4, Canada

**Keywords:** valorization, lanthanides, recycling, waste, reactive separation, electrochemistry, coprecipitation

## Abstract

SEP is an emerging green separation technique for the recovery of REE and other valuable elements from unconventional feedstocks. Its industrial adoption requires comprehensive mechanistic knowledge of its selectivity for REE vs typical background cations to achieve the desired separation. To bridge this gap, we experimentally studied SEP of neodymium Nd in chloride, nitrate, and sulfate solutions, in the absence and presence of calcium, aluminum, iron, zinc, and cobalt. We found that SEP is nonselective in the mass-transfer regime. It becomes selective in the mixed regime, with higher purification factors for elements with larger gaps in precipitation pH. At the same potential and initial pH, the selectivity of SEP in the mixed regime is controlled by the current (OH^–^ generation rate) and background ions. In the case of Fe, it additionally depends on the catalytic activity of the SEP cathode in the production of H_2_O_2_. We demonstrated for the first time that the in situ production of hydrogen peroxide in SEP can be used to selectively remove Fe from a multielement solution. The reported selectivity-recovery figures make SEP highly competitive, especially when its other advantages are factored in. The results of this study can be the basis for developing a suitable SEP-based strategy for preconcentrating REE and other valuable elements from diluted secondary resources.

## Graphical abstract

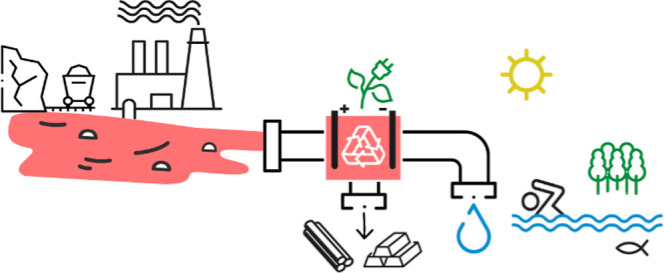

## Introduction

Rare Earth Elements (REE)—lanthanides, along with Sc and Y—are irreplaceable components of green and high tech, which makes unhindered access to them strategically critical. Even though the main source of REE remains primary ores, their virgin mining is intrinsically highly stressful for the environment, while the opening of new mines that implement more sustainable practices is very expensive and tedious. This situation has intensified interest in abundant secondary and unconventional REE resources, such as produced and acid mine/rock drainage (AMD/ARD) water, mining tailings, coal ash, REE-enriched sediments, AMD treatment sludge, battery recycling residues, and residues of bauxite, TiO_2_, and phosphoric acid production.^1–4^ In particular, AMD sources of only one mining region (the Iberian Pyrite Belt) discharge 10.7 ton/yr of light REE, 2.1 ton/yr of middle REE, 1 ton/yr of heavy REE, 3.8 ton/yr of Y, and 0.7 ton/yr of Sc.^5^ REE recovery from these resources would not only diversify REE supply but also reduce waste, thereby offering an additional environmental benefit. The zero-waste approach suggests that the recovery of REE should be combined with the recovery of other marketable elements and minerals (e.g., Cu, Co, Zn, Al, and Fe). In parallel, nonmarketable toxic elements (e.g., actinides, Pb, and As) must be removed so that decontaminated bulk residues, including water, can be repurposed or safely discharged into the environment.

### Challenges of REE Recovery from Diluted Aqueous Feedstocks

REE are extracted from wastewater and leachates of solid waste (or concentrates) using hydrometallurgical separation techniques, including chemical precipitation (neutralization, double sulfate, oxalate, and fluoride salt techniques), solid phase extraction (adsorption and ion exchange), and liquid–liquid (solvent) extraction.^6–10^ Even though these techniques can produce marketable end products, they remain economically and environmentally unsustainable especially in the case of low (<10 mg/L) and even more so ultralow (<1 mg/L) concentrations of REE in aqueous feedstocks, which typically is the case. The main reasons include

(i) huge volumes and complex, varying composition of these feedstocks,
(ii) their remote locations,
(iii) high concentrations of competing background metal ions (e.g., Ca, Mg, Fe, Al, and Zn),
(iv) the presence of organic or inorganic ligands (e.g., natural organic matter, industrial reagents, and sulfate) and suspended solids that can interfere with separation,
(v) the intrinsic technical difficulties to combine REE recovery with the recovery of other valuable elements and the removal of toxic elements such as heavy metals and actinides,
(vi) the intrinsic technical difficulties to separate individual trivalent REE.

REE recovery from dilute aqueous feedstocks typically consists of primary and secondary steps—preconcentration and purification, respectively. A typical technique used in the preconcentration step is chemical precipitation with a base, which is also called neutralization and fractional crystallization.^1,4,11,12^ The current industrial standard for purifying REEs from a preconcentrate is solvent extraction. For example, in a patented process, on site neutralization first removes Fe and Al from AMD as a disposable sludge at pH 4.0–4.5.^4,13^ Then, at pH 8.0–8.5, it produces a concentrate containing 1–5% REE, 0.4% Co, and 13% Mn, while total REE (TREE) recovery is >96%. This concentrate was dewatered and transported to a centralized facility, where it was redissolved using HCl, followed by the recovery of high-purity REE, Co, and Mn using solvent extraction. The techno-economic analysis of the centralized purification stage found its operational cost (OPEX) to be economically attractive, whereas its profitability to be defined by capital cost (CAPEX) and HCl consumption.

Unfortunately, the cost of on-site production of REE concentrate, including the management cost of sludge, has not been considered. However, this cost is significant: a benchmark OPEX + CAPEX figure for the treatment of AMD by neutralization in legacy mines is $100 AUD kL.^14^ It is predominantly driven by transport and electricity costs (mainly equipment, chemicals, pumping, and agitation),^14,15^ which originate mostly from the use of chemicals (e.g., alkali, oxidants, polymeric flocculants) and the slow/energy-expensive step of precipitate separation. Another drawback of neutralization is that it cannot provide a stable chemical environment for the nucleation and growth of precipitates. Moreover, it is technically challenging to terminate this reaction sharply at a selected point, which would allow for a higher selectivity. For example, to avoid high local pH during dosing of base solutions, a base should be dosed very slowly followed by stirring.^11^ After a precipitant is dosed into the solution, supersaturation abruptly drops as soon as precipitates are formed, which stops nucleation and induces dissolution and secondary growth processes.^16^ These processes can compromise purity by promoting coprecipitation.

Mainstream research toward more sustainable REE recovery aims to improve the operational characteristics of conventional methods and make them greener. Examples include reducing the amounts of reagents and stages used, developing more selective adsorbents and extractants, and replacing the organic phase in solvent extraction with ionic liquids. In particular, solid-phase extraction methods (ion exchange and adsorption) have been developed as alternatives to neutralization and solvent extraction. Their main advantages include simplicity and efficiency at ultralow concentrations. They operate fully in water and do not use mixer settlers or filtering equipment. Highly engineered sorbents can be impressively selective for REE vs background ions and for individual REE.^17–19^ For example, when applied to a fly ash leachate containing total REE of ∼150 μg/L (0.043 mol %), an adsorbent based on REE-selective protein (lanmodulin) increased the TREE purity in just one adsorption–desorption cycle by a factor of 2040, with the breakthrough of REEs occurring after 30 bed volumes.^17^ However, these sorbents are expensive. Moreover, the elution of chelated REE requires concentrated inorganic and/or organic acids, which impedes the on-site application of these sorbents. Finally, aqueous feedstocks depleted in REE by adsorption must be decontaminated, which increases the REE recovery cost by the high cost of conventional decontamination technologies.

At the same time, much less effort has been focused on the development of principally novel approaches, as it is much more challenging from all perspectives.

### SEP as a Promising Novel Separation Technique

An emerging technique for the recovery of valuable elements from diluted aqueous feedstocks is surface electro-precipitation (SEP).^20–24^ It removes hydrolyzable metal cations from water by precipitating them as metal (hydr)­oxides or carbonates on the surface of a cathode that electrocatalytically generates OH^–^ ions. Examples of hydrolyzable metal cations include first-row metals of the transition series in groups 4–12, Ca, Mg, Al, REE, and actinides. For a trivalent metal cation M^3+^, this reaction can be written as

1 $$M^{3+}+{3OH}^{-}={M\left(OH\right)}_{3}↓$$

The most common OH^–^-generating electrocatalytic reactions are the oxygen reduction reaction (ORR)

2 $$O_{2}+{2H}^{+}+{2e}^{-}=H_{2}O_{2},{,E}_{0}=0.695\;V\;\left(NHE\right)$$

3 $$O_{2}+{2H}^{+}+{4e}^{-}={2H}_{2}O,{,E}_{0}=1.23\;V$$

and the hydrogen evolution reaction (HER)

4 $${2H}^{+}+{2e}^{-}=H_{2},,E_{0}=0.0\;V$$

Similar to neutralization, SEP separates cations based on the pH-dependent solubility of metal (hydr)­oxides and carbonates. Precipitated cations are recovered by submerging the loaded cathode in a mildly acidic (pH 2.5–3) solution and switching the electrode potential to an anodic value.

Due to these operating principles, SEP combines advantages of electrochemical separation methods, neutralization, and solid-phase extraction methods, while overcoming their main drawbacks and providing additional cost-reducing benefits.^21,22,24^ Specifically,SEP fully operates in water. It does not rely on extraneous chemicals (acids for precipitate recovery can be produced in the anodic compartment by the oxidation of water^14^). It can be powered by renewable energy, which would further reduce its environmental footprint. SEP is simple and controllable by potential and time; hence, it can be easily automated. In contrast to nanostructured (electro)­sorbents, SEP electrodes are robust and can be fabricated from nontoxic and inexpensive materials such as carbon. In contrast to strongly complexing adsorbents, they are easily and rapidly regeneratable because of their poor chemical/specific affinity for precipitates and the instability of precipitates on positively charged electrodes at acidic pH. Finally, SEP offers an outstanding kinetic advantage in the recovery of dilute and ultradilute elements.^24^ This advantage originates from the fast precipitation of cations at the external boundary of the diffusion layer, which eliminates their slow diffusion through this layer.

As such, SEP is not novel. It has been routinely used for over a century to analyze ultradilute actinides and lanthanides as well as to deposit thin metal hydroxide films, including those of lanthanides.^20,23,25–29^ At the same time, its application to the separation/recovery of elements can be considered as novel as it has been studied in a handful of works.^22,24,30^ As far as separation of REE is concerned, Lange et al. showed in 1957 that SEP can separate Y from Sr in nitric acid.^31^ More recently, Plata and co-workers reported that SEP recovered 65% Eu after Cu was extracted by electrodeposition from a synthetic 1:1 solution of Cu and Eu.^32^ We demonstrated for the first time that SEP is capable of preconcentrating valuable elements including REE as a group from AMD.^21,22^ A further step forward was made by Su and co-workers, who developed a SEP-based technique that recovered 95% purity Ce from a Fe-depleted leachate of iron slag.^33^ Following the deposition of a mixture of elements at a cathodic potential by SEP, the electrode was polarized at an anodic potential in an acidic solution where Ce­(III) was oxidized to Ce­(IV) forming insoluble CeO_2_ while other precipitates were dissolved. However, there is currently no mechanistic understanding of the selectivity of direct SEP for trivalent REE over typical background elements.

### Selectivity of SEP

In general, impurities can partition into a precipitate via many mechanisms, including: (i) incorporation into the crystal lattice of the host crystal (precipitation of solid solutions), (ii) adsorption on the surface of the growing or already grown host crystals, (iii) mechanical entrapment into the voids of precipitates; and (iv) heterogeneous precipitation of one material on the surface/seeds of the other.^34,35^ For example, the loss of REE during the neutralization of Fe and Al at acidic pH has been explained by adsorption, with sulfate anions and basicity acting as promoters.^1,36,37^ During precipitation at pH 8, REE can also occupy iron sites in the structure of amorphous iron precipitates.^38^ In contrast, only adsorption and entrapment were observed for the REE-Al system.^39^ Partitioning is controlled by many factors such as the ratio of solubility products, the degree of supersaturation, temperature, nucleation rates, similarity of cation charge and size, as well as crystal groups of precipitates and the complex formation of the coprecipitating cations.^40–42^ Many of the partitioning reactions can be rapid, complex, and multistep, involving short-lived intermediate phases.^43^ This suggests that the precipitation kinetics also play a critical role.

Differences in the conditions of precipitate formation in neutralization and SEP can result in differences in the selectivity of these two techniques. Compared with neutralization, SEP exposes precipitates to the solution much less, both in terms of the solution volume and contact time, which can decrease the probability of impurity incorporation. In fact, SEP precipitates form compactly inside the diffusion layer and can be immediately discharged.^24^ In contrast, bulk precipitates are dispersed throughout the solution, where they are typically conditioned for several hours to grow. In addition, there is a difference in the local concentration of OH^–^ ions. In the case of SEP, the local concentration of OH^–^ ions at the precipitation front (external boundary of the diffusion layer) is almost constant as these ions are continuously supplied by the cathode. The resulting steady local supersaturation hinders aging reactions, such as dissolution and reprecipitation, and maintains a steady surface charge of the precipitates. In contrast, during neutralization, the concentration of OH^–^ ions in the micromixing region decreased abruptly as soon as these ions are consumed. Hence, precipitates are more likely to age and acquire less negative surface charge, which can affect their composition.

Finally, the production of H_2_O_2_ or H_2_ with OH^–^ by reactions 2 and 4, respectively, can affect the uptake of redox-active impurities such as Fe. Most Fe is present in AMD as Fe­(II), which starts precipitating by OH^–^ at pH ∼8.^44^ As this pH is only slightly above pH of REE precipitation, neutralization is in efficient in separating Fe­(II) from REE.^11,12^ An approach to create a precipitation window is to oxidize Fe­(II) to Fe­(III) by H_2_O_2_, which shifts Fe precipitation to pH 2.6–3.0.^11,44,45^ This technique allowed removing 97–99% of Fe from AMD at REE losses below 5%.^11,44^ However, the use of H_2_O_2_ inflates technology costs. Therefore, its in situ production by a SEP cathode can eliminate these costs. in situ production of H_2_O_2_ by a SEP cathode can be more sustainable.

Based on the above hypotheses and the promising performance of SEP in real AMD,^21,22^ this study aimed to expand the basic knowledge of the selectivity of SEP for REE vs common interfering background elements. Toward this objective, we study (i) the effect of potential on the rate-determining step of the Nd recovery by SEP, (ii) the effect of the background anion (Cl^–^, NO_3_^–^, and SO_4_^2–^) on this step, (iii) selectivity of SEP for Nd in the presence of typical co-ions such as Ca, Fe, Al, Zn, and Co, and (iv) the capability of SEP in separating Fe, Al, Zn and Co one from another. Although real feedstocks have much more complex compositions, adapting SEP to them requires an initial feasibility assessment in simpler synthetic solutions, as well as a fundamental understanding of selectivity trends. Moreover, the speciation of elements in real feedstocks is highly sensitive to fluctuations in dissolved O_2_ and CO_2_ as well as bacterial contamination during storage and handling. Along with their complex composition, this variability makes it more challenging to reproduce and interpret the trends observed in real feedstocks than in freshly prepared synthetic solutions.

Neodymium (Nd) is among the most sought-after REE. Its recovery by SEP is also relevant to water decontamination from trivalent actinides due to similar ionic radii and valence states.^46^ HCl, H_2_SO_4_, and HNO_3_ are used for leaching REE from ores and solid waste.^1,10^ Sulfate is the main anion in AMD, where its concentration can be as high as 2–50 g/L.^5,14,47–49^ Ca, Fe, and Al are the main background elements, while Zn and Co can be present in AMD of polysulfide origin. Typical Ca and Zn concentrations vary from 0.1 to 1 g/L, while concentrations of Fe and Al can reach hundreds of g/L.^5,50–52^ Concentrations of Co are much lower, within the mg/L range.^5,21,22^ All five background elements interfere with REE recovery by solvent extraction and chemical precipitation.^11,12,17,45^ Moreover, Fe, Al, Zn and Co must be removed from the remaining solution because of their toxicity to living organisms. At the same time, these elements (especially Co) have a market value. Hence, their recovery can make the REE recovery more sustainable.

## Materials and Methods

### Materials

The reagents used include Na_2_SO_4_ (anhydrous, ertified ACS, Fisher); NaNO_3_ (p.a. Merck), NaCl Sigma (BioXtra, >99.5%), and an Nd 1000 mg/L AAS standard solution in 5% HNO_3_ (Specpure, Alfa Aesar). Fe­(II), Al, and Zn were ICP & ICP–MS standards from Inorganic Ventures.

Reticulated vitreous carbon (RVC) foam panels with a porosity of 100 ppi and a relative density of 3% were purchased from Duocel. All glassware and plastic ware were washed with Sparkleen detergent (Fisher) and rinsed several times with Nanopure water. All solutions were prepared before the tests using Nanopure water with a resistivity of 18.2 MΩ cm. Their pH was adjusted using solutions of NaOH, HCl, HNO_3_, and H_2_SO_4_ (Certified ACS Plus, Fisher).

### Uptake and Regeneration

SEP tests were conducted in the batch mode using a cylindrical two-compartment glass cell.^21,24^ The cell had a Pt counter electrode, RVC working electrode, Ag/AgCl (3 M NaCl) reference, pH probe, and anion-conducting membrane separating the counter and working electrode compartments with a volume of 5 mL and 30/40 mL, respectively. The potential was controlled using a PGSTAT128N potentiostat (Metrohm) with NOVA 1.10.4. Zero time corresponds to the moment when the potential is applied. The electrodes were regenerated by electrolysis at +0.8 V in 0.1 M NaCl at pH 2.5–3.0 for 10 min, followed by washing with water.

All tests were conducted in air-saturated solutions at room temperature (23 ± 2 °C) on regenerated RVC electrodes at a stirring rate of 1200 rpm. As the pH of the solutions increased during SEP, we report both initial and temporal pH values. The potential is reported on the Ag/AgCl scale.

The Nd precipitates were characterized using XRD, SEM, FTIR, and XPS, as described in Supporting Information.

For elemental analysis, 1 mL aliquots were taken from the cell under stirring conditions at selected time intervals. These aliquots were immediately diluted 1:1 (v) with 4% v/v HNO_3_ and stored in polypropylene test tubes. Elemental concentrations were measured using an iCAP 7200 inductively coupled plasma optical emission (ICP–OES) spectrometer from Thermo at the University of Calgary. Blanks were measured for each set of samples and were subtracted from the total signal. Concentrations were calculated using external calibration curves measured in the marched matrix for each sample set. Each sample was measured in triplicate, and the average values were used. All kinetic curves were duplicated, with differences not exceeding 10%. Typical reproducibility is shown in Supporting Information of ref 22.

### Metrics

Recovery (uptake) [%] is calculated as

5 $$uptake\left(t\right)=\left(1-C\left(t\right)/C_{0}\right)\cdot 100$$

where *C*(*t*) and *C*_0_ are the time-dependent and initial mass concentrations (mg/L) of the element in the solution, respectively.

Selectivity to element Y vs element X is characterized using the purification factor β_Y/X_

6 $$\beta_{Y/X}=\frac{{C\left(Y\right)}_{c}/{C\left(X\right)}_{c}}{{C\left(Y\right)}_{0}/{C\left(X\right)}_{0}}$$

where *C*(Y)_c_ and *C*(X)_c_ are the mass concentrations of elements Y and X in the concentrate, respectively, *C*(Y)_0_ and *C*(X)_0_ are the mass concentrations of elements Y and X in the feed, respectively.

The specific recovery rate, SRR (L·min^–1^·(g of RVC)^−1^), is determined as

7 $$SRR\;\left[L\cdot {\left(g\;RVC\right)}^{-1}{\cdot \min}^{-1}\right]=m/\left(C_{0}\cdot W_{RVC}\cdot t_{linear}\right)=\left({uptake}_{linear}\cdot V^{*}\right)/t_{linear}$$

where *m* = uptake_linear_·*C*_0_·*V* is the weight (mg) of the metal removed from the solution during the initial (almost) linear segment of the kinetic curve, *t*_linear_ (min) is the time span of this segment, *C*_0_ (mg/L) is the initial concentration of the metal ion, *V* (L) is the volume of the solution in the cell, *W*_RVC_ (g) is the weight of the RVC sheet, and V* = *V*/*W*_RVC_ (L·g^–1^) is the specific solution volume, which is inversely related to the adsorbent dose *W*_RVC_/*V*.

## Results and Discussion

### SEP of Nd in NaCl Solutions

We began by studying the rate of SEP of Nd in NaCl solutions to use these results as a benchmark for further studies on the effect of the background salt anion and precipitable cations. Understanding the SEP of REEs in NaCl is also essential for optimizing this method for extracting REEs from seawater and HCl leachates. The study is conducted at an Nd concentration of 10 mg/L, which is within the upper limit reported for REE in AMD and leachates.^11,17,21,22,39^

Figure 1a shows the kinetics of the Nd uptake at an initial concentration of 10 mg/L and initial pH 7.0 in 0.1 M NaCl at −0.2 V, −0.3 V, and −0.4 V. At all three potentials, the kinetic curves overlapped, reaching a 95% plateau within 9 min. During these tests, the pH of the bulk solution increases to 10.0–10.7, with both the rate and the final value being higher at a more negative potential (Figure 1b). It follows that SEP rate under the above conditions does not depend on pH (OH^–^ generation rate). It also remains the same when the background NaCl concentration decreases from 0.1 to 0.01 M (Figure 1a).

**Figure 1:**
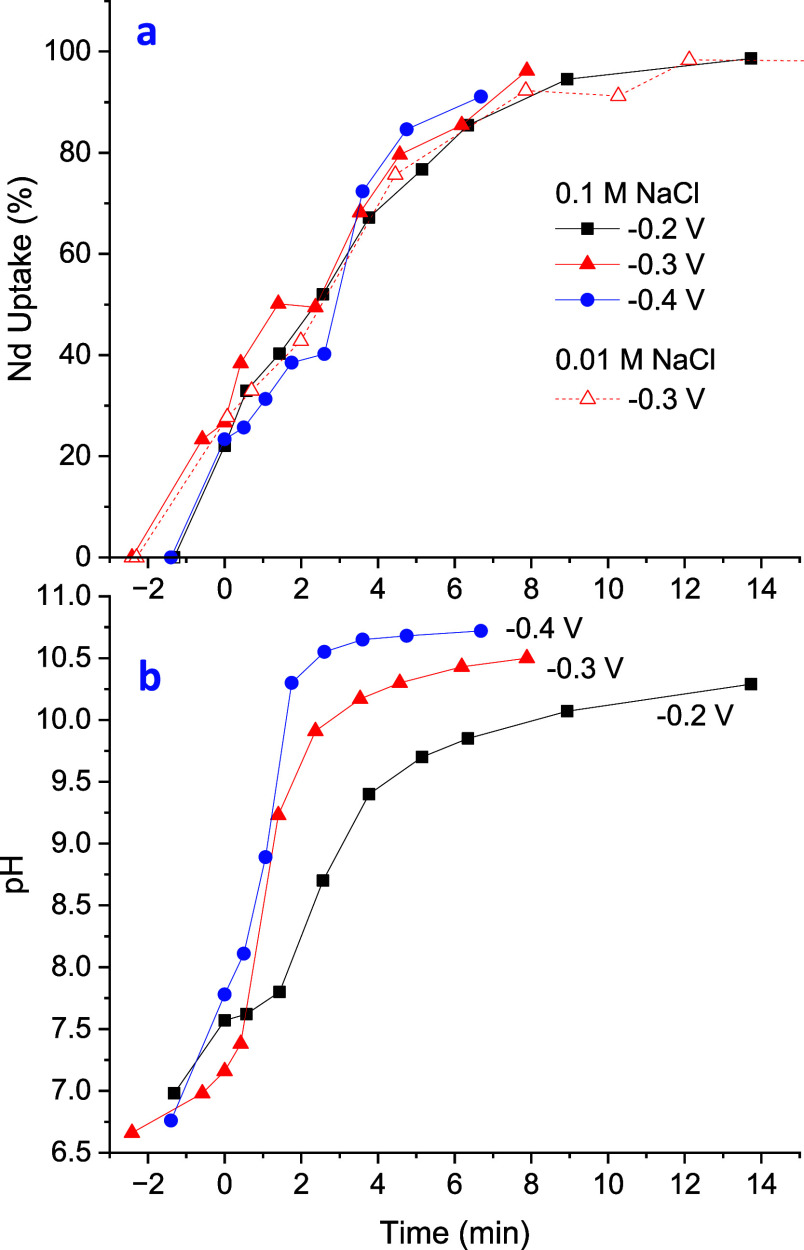
Effect of potential and NaCl concentration on SEP of Nd at 10 mg/L Nd and initial pH 7.0: (a) the Nd uptake at −0.2 V, −0.3 V, and −0.4 V in 0.1 M NaCl as well as at −0.3 V in 0.01 M NaCl (b) corresponding pH of the bulk solution in 0.1 M NaCl. The cell volume is 40 mL. The RVC electrode size is 1.7 × 0.9 × 0.32 cm^3^ (0.038 g). pH 7.0 is set in the stock solution. In the cell, it dropped to pH ∼ 6.7 due to Nd adsorption on the RVC electrode. The error bar for the kinetic curves is below 10%.

The insensitivity to the electrode potential (pH) and salt concentration indicates that the SEP rate is controlled by the mass transfer rather than the kinetics of precipitation.^24^ In the mass-transfer regime, SEP rate (SRR) is proportional to the specific solution volume multiplied by the rate with which the precipitable element is transferred to the external boundary of the diffusion layer.^24^ The mass-transfer controlled SRR of Nd in this system is 0.14 ± 0.02 L·(g RVC)^−1^·min^–1^. This value is close to the mass-transfer-controlled SRR of Pb.^24^ Hence, as expected based on the mass-transfer mechanism,^24^ SEP does not exhibit selectivity in the mass-transfer regime, which is confirmed by further tests in multielement solutions (see below).

The Nd precipitate formed by SEP at −0.3 V in 0.01 M NaCl consisted of polydisperse shapeless particles (Figure S1). They were structurally amorphous, as indicated by the absence of Bragg peaks in their X-ray diffractogram (Figure S2). Amorphous precursors with compositions depending on the precipitation conditions are typically observed for trivalent lanthanides in (bi)­carbonate solutions.^53–55^ These precursors transform to stable crystalline phases in several days or even weeks.^53–55^

The chemical composition and structure of the amorphous Nd precipitates in 0.01 M NaCl were identified using XPS and FTIR. XPS showed that the precipitate surface contained (bi)­carbonate groups with a C/Nd atomic ratio of 1.8 (Figure S3). Because we were unable to obtain FTIR spectra of the precipitate due to its low amount, we measured FTIR spectra of a bulk precipitate formed by the rapid titration of an air-saturated 10 mg/L Nd solution in 0.01 M NaCl with 0.1 M NaOH up to pH 9.5. These spectra show that the precipitate consisted of hydrated normal carbonate Nd_2_(CO_3_)_3_·*n*H_2_O rather than hydrated basic carbonate (hydroxycarbonate) Nd­(OH)­CO_3_·*n*H_2_O or hydroxide Nd­(OH)_3_·*n*H_2_O (Figure S4). The formation of normal carbonate agrees with the XPS results and general knowledge that carbonates of trivalent lanthanides are more stable (less soluble) than hydroxides.^12,56^

Accordingly, speciation modeling using Minteq 3.1 predicts that a 10 mg/L Nd solution in 0.01 M NaCl in the absence and presence of atmospheric CO_2_ becomes saturated with respect to Nd­(OH)_3_ and Nd_2_(CO_3_)_3_ at pH 8.3 and 6.9, respectively (Figure S5d). The precipitation pH of the metastable amorphous Nd carbonate is likely to be much lower than pH 6.9, as follows from the comparison of the SEP rates for Nd and Pb in 0.01 M NaCl. As shown earlier, Pb precipitates in SEP as crystalline hydrocerussite Pb_3_(CO_3_)_2_(OH)_2_.^24^ Under the same solution conditions, its theoretical precipitation pH is 6.5, suggesting that Pb would precipitate faster than Nd. However, the opposite is observed in SEP. At −0.2 V in 0.01 M NaCl, SEP of Pb is still in the mixed (mass-transfer-kinetic) regime,^24^ while SEP of Nd is already in the mass-transfer regime (Figure 1). Since the mass transfer rates for Pb and Nd are similar (see above), this fact suggests that Nd precipitates faster than Pb. The discrepancy with the theoretical relationship can be explained by the metastability of amorphous Nd_2_(CO_3_)_3_·*n*H_2_O, which results in its formation at lower pH compared to crystalline Nd_2_(CO_3_)_3_. Hence, in the following, in lieu of the stability constants of metastable phases, we use thermodynamically predicted precipitation trends with caution, only when they accurately describe the experimentally observed trends.

In this context, we should note that the transformation of metastable precursors during the precipitation of inorganic compounds is among the main differences between classical and nonclassical nucleation theories, which are currently a matter of debate.^57^ The former describes a precipitation pathway using traditional thermodynamics of phase coexistence and assumes the addition of monomeric species to nuclei and seeds. In contrast, the nonclassical theory considers precipitation precursors ranging from stable prenucleation clusters to liquid (nano)­droplets and/or (nano)­particles. The above analysis suggests that the potential at which SEP kinetics switch from the mixed regime to the mass-transfer regime can be used as a novel tool for rapid qualitative ranking of the kinetics of precipitate formation for different metal cations or the same cation in different solutions (see also section “Selectivity of SEP for Nd vs Ca”), which may help further the debate about the precipitation mechanism.

### Effect of Anions on SEP of Nd

Another important characteristic of SEP that remains unknown is the sensitivity to background anions. As shown in Figure 2, the Nd uptake rate in 10 mg/L Nd solutions at −0.3 V decreases in the order of NaCl > NaNO_3_ > Na_2_SO_4_. The decrease in the SEP rate by nitrate and further by sulfate suggests that these anions retard the precipitate formation, thereby switching SEP from the mass-transfer to mixed regime. In the mixed regime, the SEP rate depends not only on the supply rate of cations to the external boundary of the diffusion layer but also on the supersaturation at this boundary. Anions can affect supersaturation through the formation of soluble ionic pairs or complexes with precipitable cations, which changes the concentration of free ions that enter the expression for the solubility product equilibrium constant. In addition, anions can affect the crystal growth through adsorption on the crystal facets/defects.^58^ Finally, they can affect the catalytic activity of the SEP cathode in the OH^–^ generation through competing with OH^–^/H_2_O for the catalytic sites.^59^

**Figure 2:**
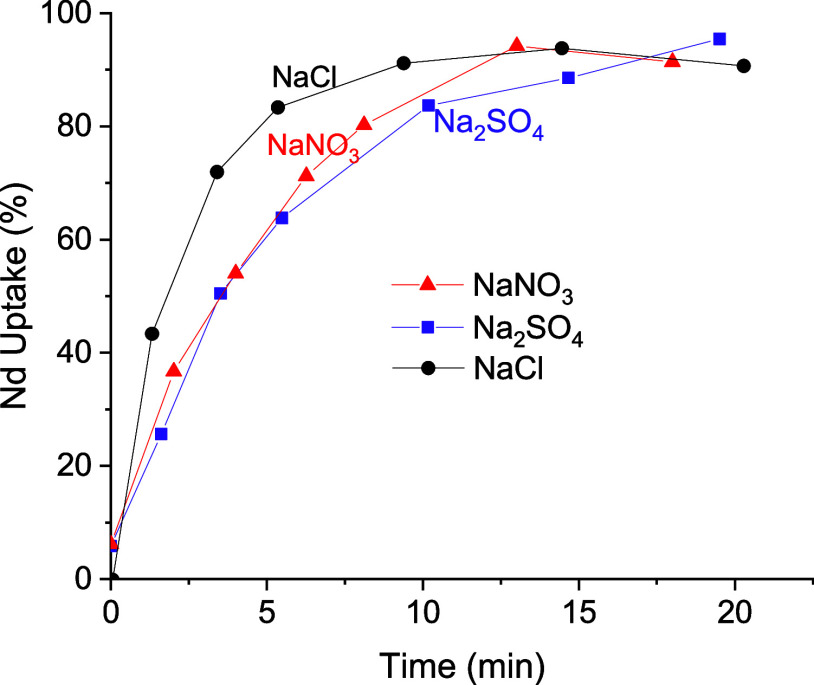
Effect of background anions on SEP of Nd at −0.3 V in 10 mg/L Nd solutions in 0.01 M NaCl, NaNO_3_, and Na_2_SO_4_ at initial pH 7. The cell volume is 30 mL. The RVC size is 1.7 × 1.7 × 0.32 cm^3^ (0.065 g). The error bar is below 10%. The corresponding current is shown in Figure S8.

Given that the SEP current and, hence, local pH increase in the opposite order (NaCl < NaNO_3_ < Na_2_SO_4_) (Figure S8) compared to that of SEP rate, the decrease in the SEP rate is likely caused by the increased stability of the soluble Nd-anion complexes. The stability of Ln^3+^-anion complexes increases in the order of Cl^–^ < NO_3_^–^ < SO_4_^2–^ < OH^–^ < CO_3_^2–^.^49,60–62^ Both SO_4_^2–^ and NO_3_^–^ form inner-sphere complexes, while Cl^–^ preferably forms outer-sphere 1:1 complexes (contact ion pairs are formed only at high Cl^–^ concentrations).^63,64^

The mixed regime of SEP at −0.30 V in the sulfate solution is confirmed by a decrease in SEP rate with an increase in sulfate concentration from 0.01 to 0.0345 M (Figure 3a). This effect is underpinned not only by the anion-Nd complexation but also by the OH^–^ generation mechanism: both the current and bulk pH decrease at 0.0345 M Na_2_SO_4_ (Figures S9 and 3b, respectively), contributing to a decrease in supersaturation. The mixed regime at −0.3 V is also supported by the increase in the SEP rate at −0.75 V (Figure 3a). At −0.75 V, SEP is in the mass-transfer regime as follows from the independence of the SEP rate from the sulfate concentration and bulk pH (Figure 3). The SRR of Nd at −0.75 V in Na_2_SO_4_ is 0.16 L·(g RVC)^−1^·min^–1^, which is close to that of Nd in NaCl in the mass-transfer regime. Hence, the SEP in this regime is much less sensitive to background anions than in the mixed regime.

**Figure 3:**
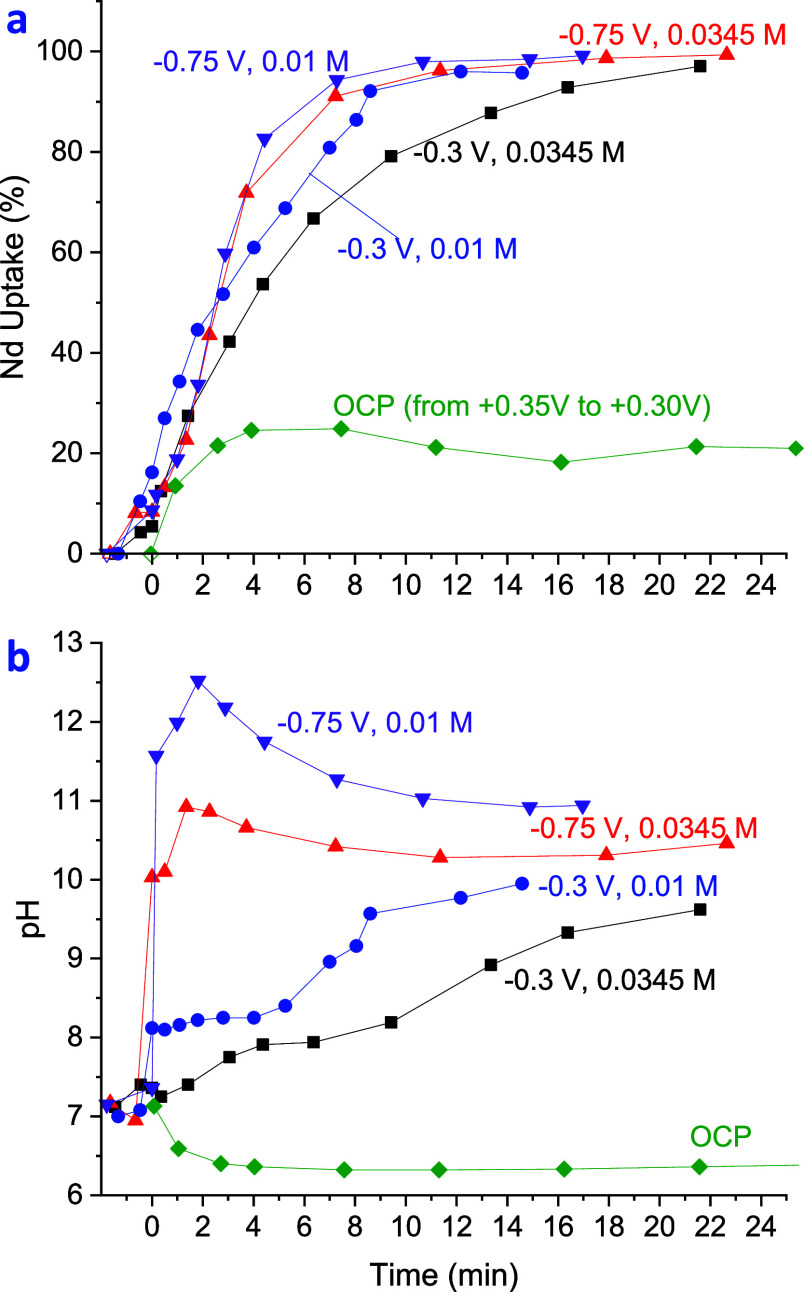
Time dependence of SEP at −0.30 V and −0.75 V and adsorption at open circuit potential (OCP) in a 10 mg/L Nd^3+^ solution in 0.01 and 0.0345 M Na_2_SO_4_ at initial pH 7.0. (a) Nd uptake and (b) corresponding bulk solution pH. The cell volume is 40 mL. The RVC size is 1.7 × 0.9 × 0.32 cm^3^ (0.038 g). OCP = open-circuit potential. The error bar for the kinetic curves is below 10%.

Finally, the adsorption of Nd on RVC at the open-circuit potential (OCP) in 0.0345 M Na_2_SO_4_ uptakes only 22 ± 2% Nd, which is reached in 4 min (Figure 3a). In contrast to SEP, adsorption decreases pH from 7.0 to 6.2 (Figure 3b). This effect can be ascribed to the cation exchange of Nd­(SO_4_)^+^, which is the main Nd species in the sulfate solution at neutral pH (Figure S7a), with protons on the oxidized RVC surface. The SRR of the Nd uptake by adsorption of 0.058 L·(g RVC)^−1^·min^–1^ is by a factor of 2.5 lower than that of SEP in the mass-transfer regime. This result further confirms the unique capability of SEP to overcome the diffusion barrier as observed in our previous study with Pb^2+^ ions.^24^

In summary, background anions can switch the rate-determining step of SEP from the mass-transfer regime to the mixed regime. In the mass-transfer regime, SEP rate is not sensitive to background anions. It becomes sensitive to them in the mixed regime. The dependence of the SEP regime on background anions is important, as the mixed regime is beneficial for selectivity (see below), while the mass-transfer regime provides a higher uptake rate.^24^

### Selectivity of SEP for Nd vs Ca

We studied the effect of Ca on SEP of Nd in 0.01 M Na_2_SO_4_ and 0.01 M NaCl at 1000 mg/L Ca and 10 mg/L Nd, that is, at a Ca/Nd molar ratio of 360. This Ca concentration is close to the upper limit reported for AMD,^50,51^ which is controlled by saturation with respect to gypsum (CaSO_4_·2H_2_O). At 1000 mg/L Ca, 0.01 M sulfate solutions are not saturated with respect to gypsum (CaSO_4_·2H_2_O) (Figure S7b).

As shown in Figure 4a, the 360× excess of Ca hardly affects the Nd uptake at −0.30 V in 0.01 M Na_2_SO_4_ at initial pH 7. The exception is the first 2 min, when the uptake in the presence of Ca is by 5–10% lower. The inhibiting effect of Ca is somewhat more pronounced at −0.75 V, where the Nd recovery rate is reduced by Ca by 10% (Figure 4b). In contrast, the inhibiting effect of Ca on the SEP rate becomes apparent in 0.01 M NaCl (Figure 4c).

**Figure 4:**
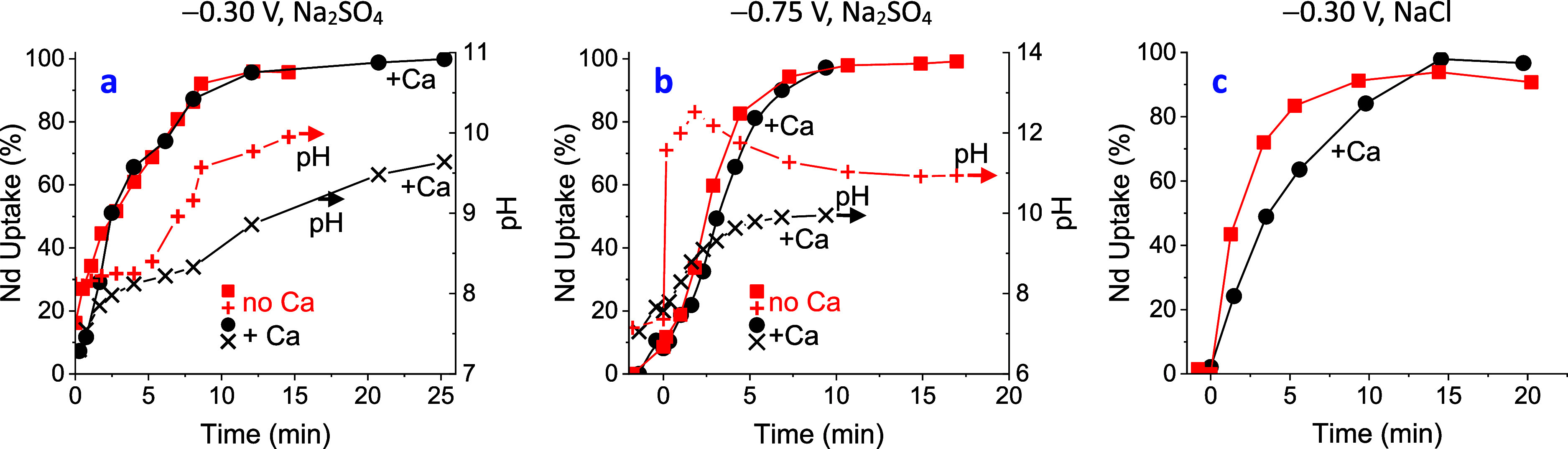
Effect of 1 g/L Ca on SEP of Nd in 0.01 M (a,b) Na_2_SO_4_ and (c) NaCl, initial pH 7.0, at (a,c) −0.30 V and (b) −0.75 V. Ca is added as CaCl_2_. (a,b) The cell volume is 40 mL. The RVC size is 1.7 × 0.9 × 0.32 cm^3^ (0.038 g). (c) The cell volume is 30 mL. The RVC size is 1.7 × 1.7 × 0.32 cm^3^ (0.065 g). The corresponding current is shown in Figure S10. The error bar for the kinetic curves is below 10%.

The inhibiting effect of Ca on the Nd uptake rate is likely to be caused by the passivation of the RVC surface by CaCO_3_, given that Ca inhibits both the pH increase and the OH^–^ current (Figure 4a,b and S10, respectively). Theoretically, CaCO_3_ is expected to precipitate at pH above 7.6 (Figure S7b). Hence, the stronger effect of Ca at −0.75 V compared to −0.30 V can be explained by the higher actual pH at −0.75 V. The stronger inhibiting effect of Ca in the chloride solution than in the sulfate solution can be explained by the stronger precipitation of CaCO_3_ in the former. Although both Cl^–^ and SO_4_^2–^ inhibit CaCO_3_ precipitation, SO_4_^2–^ does it more strongly.^65,66^ As a result, the Nd/Ca selectivity of SEP is expected to be higher in sulfate solutions than in chloride solutions.

We can only estimate the lowest limit of the Nd/Ca purification factor β_Nd/Ca_ because the Ca concentration remains the same within the accuracy of the ICP–OES measurements (<5%). Assuming that 50 mg/L (5%) Ca is coprecipitated with 9.9 mg/L Nd, the lowest (most conservative) estimate of β_Nd/Ca_ at 99% Nd recovery in 0.01 M Na_2_SO_4_ is ∼10^2^. A similar β_TREE/Ca_ value was reported for SEP at −0.75 V in AMD at a 99% REE recovery and final pH 8.7.^22^ At the same time, almost full Ca rejection was detected in AMD at −0.75 V in 3.2 min, where 70% REE was recovered and bulk pH reaches 7.5.^22^

To compare, the one-stage neutralization of AMD at pH 7.45 with NaOH recovered 99% REE at β_TREE/Ca_ of 43, which is twice as low as that in SEP at the same recovery (Table S1 in ref 22). A β_TREE/Ca_ of ∼50 characterized the REE concentrate produced from AMD by neutralization at pH 8–8.5 after Fe and most Al were removed at pH 4–4.5.^13^ Much lower β_TREE/Ca_ of 10.6 and 3.3 were reported for, respectively, the single-stage supported liquid membrane and solvent extraction in fly ash leachates that had only 40–50× excess of Ca over REE.^68^ The higher Nd/Ca selectivity of SEP and neutralization, even at pH above 7.6 (at supersaturation with respect to CaCO_3_), can be linked to the known inhibiting effect of even trace REE amounts on CaCO_3_ crystallization.^67^ However, in the case of SEP, there is an additional contribution of the intrinsic SEP features, as the Nd/Ca selectivity of SEP is twice as high as that of neutralization.

In contrast to SEP of Nd, SEP of Pb is promoted by Ca at −0.20 V in 0.01 M NaCl.^24^ Considering that SEP of Pb at −0.20 V in 0.01 M NaCl is in the mixed regime,^24^ the difference can be attributed to the promoting role of calcite seeds on the precipitation Pb. Calcite has strong affinity for Pb, resulting in the fast surface precipitation of Pb–Ca carbonate on its surface.^69,70^

In summary, SEP demonstrates high selectivity for Nd vs Ca at a Ca/Nd molar ratio of 360. The partitioning of Ca to the precipitate is controlled by the background anions, being lower in sulfate solutions than in chloride solutions. It is also controlled by the interaction of Nd with CaCO_3_. The sensitivity of SEP to these interactions under dynamic conditions makes this technique a novel tool for studying them.

### Selectivity of SEP for Nd vs Al, Fe, Zn, and Co

The separation of Fe, Al, Zn, and Co from Nd was studied at concentrations of 2.5, 5, and 10 mg/L, as these concentrations are within the range reported for AMD and leachates.^5,21,22,50–52^ Even though the Fe and Al concentrations in AMD and leachates can be much higher, at higher concentrations these elements rapidly passivate RVC electrodes. The theoretical precipitation pH at 10 mg/L of these elements in 0.01 M NaCl increases in the order: Fe­(III) (2.7 for ferrihydrite) < Al (4–5 for amorphous Al­(OH)_3_ and boehmite) < Nd (6.9 for Nd carbonate) < Zn (∼7.5 for Zn­(OH)_2_) < Fe­(II) (7.5 for siderite) < Co (8.2 for Co­(OH)_2_) (Figure S5). The closer the precipitation pH of the two elements is, the more difficult it is to separate them.

Figure 5a shows that the Nd/Fe selectivity at −0.3 V in a binary Nd + Fe­(II) solution in 0.01 M NaCl is poor. The initial Nd and Fe­(II) concentrations are 10 mg/L each and initial pH is 4.4. The largest uptake difference is observed between 2 and 8 min, during which approximately 30% more Nd is recovered than Fe, while the bulk pH remains below 6. The Fe uptake initially experiences a ∼5 min delay vs Nd, while the uptake slope (and, hence, the specific uptake rate SRR) for these two elements is similar. The selectivity vanishes after 13 min, when bulk pH reaches 9, and both elements are almost completely removed. Considering the trend in precipitation pH (see above), we conclude that SEP uptakes Fe in this system mostly as Fe­(II). This conclusion is supported by the trends in neutralization with NaOH:^11^ It has a relatively poor Nd/Fe selectivity in a REE + Fe­(II) + Al solution. Selectivity significantly increases in the presence of H_2_O_2_ because Fe­(III) precipitates at much lower pH than Nd.^11^

**Figure 5:**
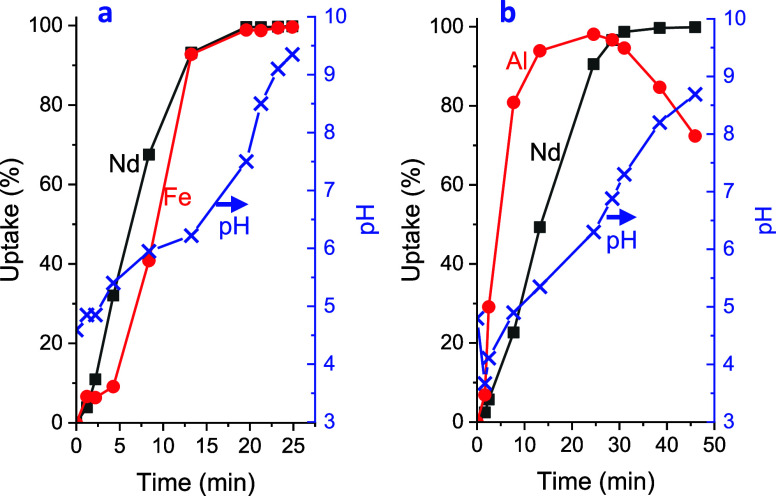
SEP at −0.3 V in binary solutions of Nd with (a) Fe and (b) Al, 10 mg/L each, in 0.01 M NaCl at initial pH 4.4 and 4.8, respectively. Fe is added as Fe­(II) nitrate. The cell volume is 40 mL. The RVC size is 1.7 × 0.9 × 0.32 cm^3^ (0.038 g). The corresponding current is shown in Figure S11. The error bar for the kinetic curves is below 10%.

The uptake of Fe as Fe­(II) suggests that H_2_O_2_ production during SEP in the binary Nd + Fe­(II) solution is sluggish. This can be attributed to passivation of the RVC cathode by ferric (hydr)­oxides. The latter is known as a catalyst of the 4-electron ORR reaction 3,^71^ in contrast to RVC which catalyzes at −0.3 V the 2-electron ORR reaction 2.^24^

The SEP selectivity in the binary Al + Nd solution is significantly higher than in the binary Nd + Fe­(II) solution. At 7.7 min, when bulk pH reaches 4.9, SEP uptakes 81% Al and 23% Nd (Figure 5b). The uptake rate for Nd in the presence of Al is four times lower than that in the Al-free solution (Figures 5b and 3a, respectively). The corresponding purification factor β_Nd/Al_ in the residual solution is 4.0 at the 77% Nd recovery. Afterward, the Al/Nd selectivity decreases and then increases again because the Nd recovery increases, reaching a 99% plateau at 31 min, while Al recovery reaches a maximum of 98% at 25 min. The increase in Al in the solution after 25 min can be explained by the instability of Al (hydr)­oxides against Al­(OH)_4_^–^ ions at a basic pH. In this region, the Al and Nd uptakes are decoupled one from another, indicating that at a basic pH these two elements precipitate independently, rather than one pulls another by one of the coprecipitation mechanisms mentioned in the Introduction. Bulk pH increases more slowly in the Al + Nd solution than in the Fe + Nd solution (Figure 5 and Table 1), which can be attributed to the passivating character of Al (hydr)­oxide precipitates.^22^ As a result, Al switches SEP from the mass-transfer regime to the mixed regime, which boosts selectivity.

**Table 1:** Maximum Selectivity (Purification Factors) and Bulk pH after 15 min of the SEP Tests Shown in Figures 5 and 6

background solution, SEP potential	concentrations of elements, initial pH	bulk pH after 15 min of SEP	β_Nd/Al_ and β_Nd/Fe_ purification factors
0.01 M NaCl, –0.3 V	Nd + Al, 10 mg/L each, pH 4.4	5.5	β_Nd/Al_ = 4 in the residual solution at a 77% Nd recovery
	Nd + Fe(II), 10 g/L each, pH 4.8	6.5	β_Nd/Fe_ = 1.7 in the precipitate at 67% Nd recovery
	Nd + Al + Fe(II) + Zn, 10 mg/L each, pH 4.5	4.8	β_Nd/Al_ and β_Nd/Fe_ = 20 in the residual solution at a 87% Nd recovery
	Nd + Al + Fe(II) + Zn, 2.5 mg/L each, pH 4.75	6.7	no selectivity
0.01 M Na_2_SO_4,_ –0.75 V	Nd + Al + Fe(II) + Zn, 10 mg/L each, pH 3.3	4.3	β_Nd/Al_ = 4, β_Nd/Fe_ = 12 in the residual solution at a 95% Nd recovery

Importantly, the selectivity of SEP for Fe and Al vs Nd is significantly improved when Fe and Al are present together (Figure 6a and Table 1). In a 0.01 M NaCl solution of 10 mg/L Fe, Al, Nd, and Zn each, the uptake of Fe and Al gradually increases after −0.3 V is applied, reaching 96% and 92% at 17 and 40 min, respectively (Figure 6a). For Nd and Zn, the pattern is different: 13% Nd and 8% Zn are removed instantly when the potential is applied and their uptake does not change during the following 29 min, when bulk pH remains below 5.1. Afterward, the uptake of Nd and Zn starts increasing, with a faster rate for Nd than for Zn. The delay for Nd and Zn results in the almost complete removal of Fe and Al at the Nd and Zn losses of only ∼10%. The residual solution at 29 min is characterized by a purification factor β_Nd(Zn)/Fe(Al)_ of ∼20 at ∼90% Nd and Zn recovery. Hence, SEP is capable of separating Fe and Al from REE and Zn.

**Figure 6:**
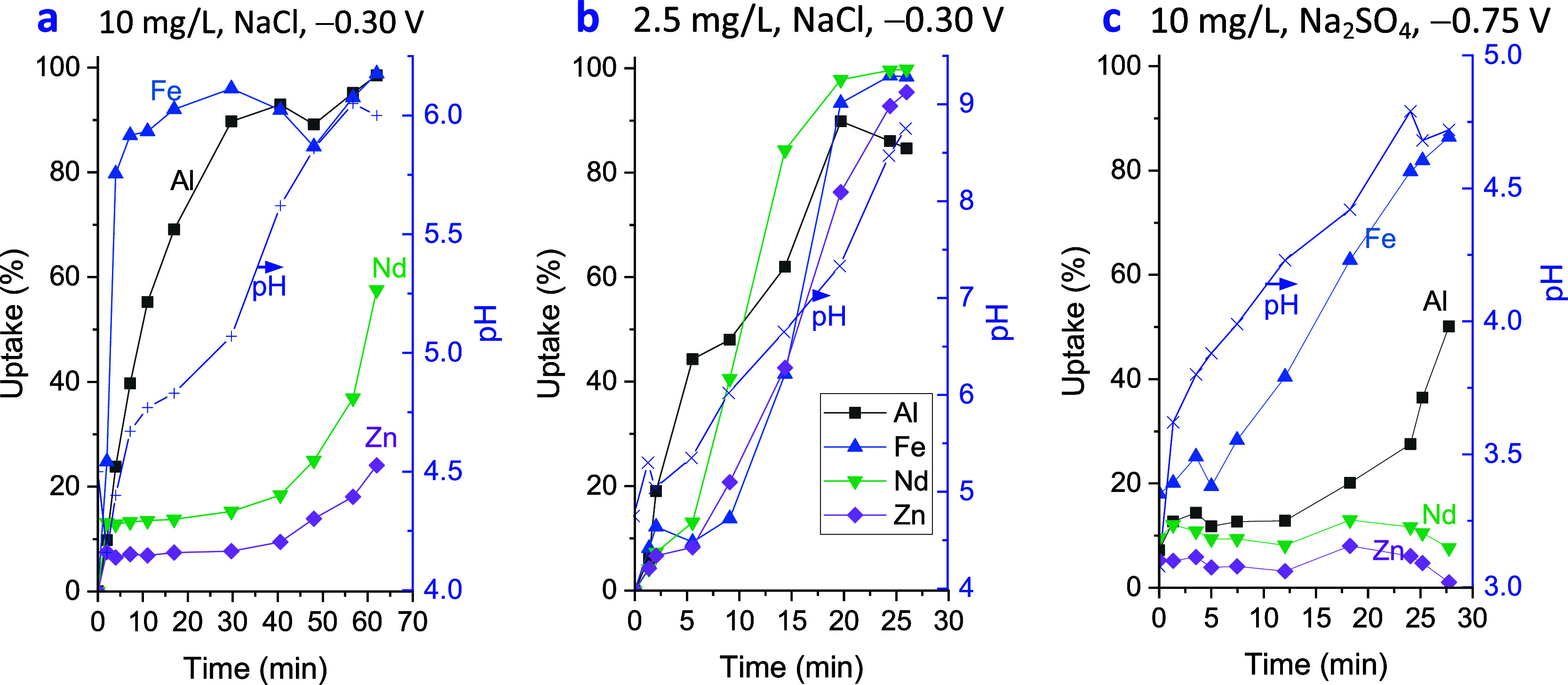
SEP in mixed-element solutions of Fe, Al, Nd, and Zn (a) at −0.30 V in 0.01 M NaCl, pH 4.5, (b) at −0.30 V in 0.01 M NaCl pH 4.75, and (c) at −0.75 V in 0.01 M Na_2_SO_4_, pH 3.3. Concentration of each element is (a,c) 10 mg/L and (b) 2.5 mg/L. Fe is added as Fe­(II) nitrate. The cell volume is 40 mL. The cell volume is 40 mL. The RVC size is 1.7 × 0.9 × 0.32 cm^3^ (0.038 g). The error bar for the kinetic curves is below 10%.

The instant initial uptake of Nd and Zn and its invariability during the following 29 min indicate that at pH below 5.1 these two elements are adsorbed on RVC through a purely capacitive (electrostatic) mechanism. The increase in the uptake of Nd and Zn after 29 min, when bulk pH increases from 5.1 to 6.0, is caused by SEP. The higher uptake rate for Nd than for Zn is consistent with their thermodynamic precipitation pH of 6.9 and 7.6, respectively (Figure S5).

The 8-fold decrease in the Fe uptake rate at −0.75 V (Figure 6c) vs −0.30 V (Figure 6a) confirms that the Fe uptake is controlled by the H_2_O_2_ production rate. Even though the OH^–^ production rate at −0.75 V is much higher than at −0.30 V (Figure S10), no H_2_O_2_ is produced at −0.75 V where the cathodic reaction 4 produces H_2_.^24^ Moreover, generated H_2_ can act as a reductant, which would suppress Fe­(II) oxidation by dissolved oxygen.

SEP loses its selectivity when the concentrations of the four elements are decreased to 2.5 mg/L (Figure 6b). After 15 min, bulk pH increased to 6.8, compared to 4.8 at 10 mg/L (Table 1). Given that the OH^–^ generation rate increases with the reactive surface area of the cathode, the rapid pH increase at 2.5 mg/L suggests its lower coverage by passivating precipitates. As a result of high local pH, SEP operates in the mass-transfer regime, where all four elements precipitate at the same rate.

Given that SEP can separate Nd and Zn from Fe and Al (Figure 6a,c), it is of interest to find out whether it can separate Nd from Zn. Figure 7a shows that SEP at −0.30 V does have this capability. In a binary Nd + Zn solution in 0.01 M NaCl containing 5 mg/L Nd and 10 mg/L Zn at pH 5, SEP uptakes Nd much faster than Zn. At 97% Nd recovery, 24% of Zn coprecipitates, resulting in a β_Nd/Zn_ value of 4. This selectivity is better than that achieved by neutralization in AMD, where 45% Zn coprecipitated with 99% REE at pH 7.^11^

**Figure 7:**
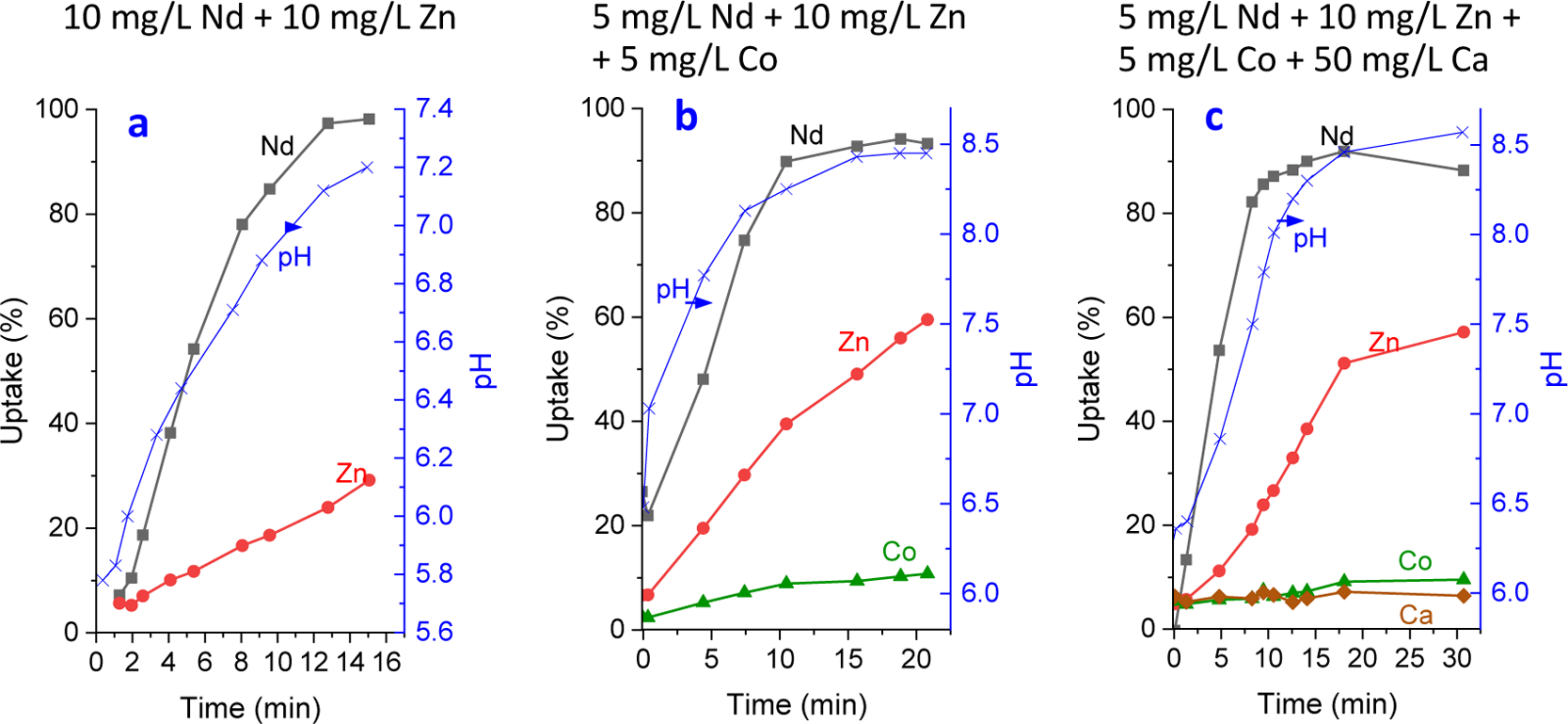
SEP at −0.30 V in mixed-element solutions in 0.01 M NaCl of (a) Nd (5 mg/L) and Zn (10 mg/L) at initial pH 5, (b) Nd (5 mg/L), Zn (10 mg/L), and Co (5 mg/L) at initial pH 6, (c) Nd (5 mg/L), Zn (10 mg/L), Co (5 mg/L), and Ca (50 mg/L) at initial pH 6. The cell volume is 40 mL. The RVC size is 1.7 × 0.9 × 0.32 cm^3^ (0.038 g). The error bar for the kinetic curves is below 10%.

SEP is also capable of separating Nd and Zn from Co. In a Nd + Zn + Co solution in 0.01 M NaCl, at 5 mg/L Nd, 10 mg/L Zn, and 5 mg/L Co, at initial pH 6, the SEP at −0.30 V uptakes 90% Nd, 40% Zn and 9% Co in 10 min (Figure 7b), while 50 mg/L Ca does not affect this separation (Figure 7c). The uptake of Co remains suppressed even when bulk pH reaches 8.5. The corresponding Nd/Co purification factors β_Nd/Co_ are 9.5–10. To compare, 70% Co coprecipitates with 99% REE at pH 7.^11^ Hence, due to the different precipitation conditions, SEP can be more beneficial for separation than neutralization.

Thus, the selectivity of SEP is generally controlled by the rate of OH^–^ generation (SEP current). The selectivity for Fe is additionally controlled by the H_2_O_2_ production rate. For the same SEP cathode, both rates depend on the potential of the cathode and its coverage by precipitates. Passivation can switch SEP from the mass transfer regime to the mixed regime, which underpins the selectivity of SEP in general. Precipitation of Fe (hyrd)­oxides suppresses H_2_O_2_ production. In the Nd/Zn and Nd/Co separations, SEP demonstrated better selectivity at similar recoveries than neutralization with NaOH. Even though the Fe/Nd selectivity of SEP in our tests is comparable to that of neutralization with H_2_O_2_ and NaOH,^11^ it is achieved without using any reagent and much faster. Further optimization of SEP is required to improve its selectivity.

## Conclusions

Neodymium precipitates in NaCl solutions as amorphous normal carbonate, which suggests a critical role of partial CO_2_ pressure and mixing conditions in the recovery of REE by SEP. The metastability of the precipitated Nd carbonate suggests that thermodynamic speciation modeling should be used with caution to predict SEP trends involving Nd precipitation.

At −0.30 V in 10 mg/L Nd solutions in 0.01 M NaCl, SEP is in the mass-transfer regime, but switches to the mixed regime in 0.01 M NaNO_3_ and 0.01 M Na_2_SO_4_. As a result, the SEP rate at −0.30 V decreases in the order NaCl > NaNO_3_ > Na_2_SO_4_, which is explained by the Nd-anion complexation mechanism.

SEP demonstrates competitively high Nd/Ca selectivity (β_Nd/Ca_ > 100), which is higher in sulfate solutions than in chloride solutions. Its high Nd/Ca selectivity can be explained by the inhibiting effect of Nd on CaCO_3_ precipitation interwined with the specific conditions of precipitate formation.

SEP can achieve high selectivity in the separation of Fe and Al from Nd and Zn at potentials where H_2_O_2_ is produced by the SEP cathode by reaction 2. Al promotes Fe separation from Nd. SEP is also capable of separating Nd, Zn, and Co individually from their mixtures.

These results indicate that SEP is selective when its rate-determining step is in the mixed kinetic regime but does not have selectivity in the mass-transfer regime. These regimes can be controlled by adjusting the parameters that affect the local supersaturation at the cathode-solution interface, such as background anions/ligands and the OH^–^ generation rate.

The found conditions of low and high selectivity of SEP provide a crucial missing link for the potential industrial application of this technique, helping guide its rational design. Also, SEP can serve as a rapid tool for qualitatively ranking the kinetics of precipitate formation for different metal cations as well as their coprecipitation behavior under dynamic conditions.

## Supplementary Material



## Notes

XRD, SEM, XPS, and FTIR characterization of Nd precipitate. Speciation Modeling using Visual Minteq 3.1. Chronoamperometry during SEP tests (PDF)
