# Supplementary material for: Controlling Selectivity of Surface Electro-Precipitation (SEP) in the Recovery of Rare Earth Elements (REE) from Aqueous Feedstocks
Source: ACS Sustain Chem Eng. 2025 Jun 12;13(25):9630–41. doi: 10.1021/acssuschemeng.5c02403 (PMC12219096; doi:10.1021/acssuschemeng.5c02403)
Supplement: Supplementary file 1 [file sc5c02403_si_001.pdf]

Supplementary Information

Controlling selectivity of surface electro-precipitation (SEP) in the recovery of rare earth elements (REE) from aqueous feedstocks

Irina V. Chernyshova,*[a] Wesam Tork,[b] and Sathish Ponnurangam[b]

[a] Department of Earth and Environmental Engineering, Columbia University, 10027, New York, USA. Current affiliation: US Gypsum Corporation, Libertyville, USA

[b] Department of Chemical and Petroleum Engineering, University of Calgary, Calgary, T2N 1N4 Alberta, Canada

*Corresponding author. Current e-mails: ichernyshova@usg.com, irina905C@gmail.com

Table of Content

- XRD, SEM, XPS, and FTIR characterization of Nd precipitate p.2
- Speciation Modelling using Visual Minteq 3.1 p.6
- Chronoamperometry during SEP tests p.8

Contains 10 pages and 11 figures

XRD, SEM, XPS, and FTIR characterization of Nd precipitate in 0.01 M NaCl

The crystalline phases of the Nd deposits were analyzed using Powder X-ray diffraction (XRD). Diffractograms were obtained using a Bruker D8 Advance ECO diffractometer (Bruker, Germany) with Cu−Kα radiation (λ = 1.5418 Å). A 2θ range from 10° to 60° was scanned with an increment of 0.025° per second at 2 s at each step. The phase identification was performed using complete structural reference from the Inorganic Crystal Structure Database (ICSD).

Scanning electron microscopy (SEM) imaging with energy dispersive X-Ray spectrometry (EDS) elemental analysis was performed using a Helios NanoLab 660 instrument.

X-ray photoelectron spectra (XPS) were measured using VersaProbe II with a mono Al (1486.6 eV) at 25.3 W micro-focused X-ray source at a takeoff angle of 45°. The instrument neutralizes the sample charging using a dual-beam charge compensation system that utilizes both a cool-cathode electron flood source and the very-low-energy ion capability of the Argon ion sputter gun. The diameter of the probing area is 100 mm. The pass energy for the survey and multiplex spectra is 117.4 and 23.5 eV, respectively. The calibration binding energy is defined by the Au $4f_{7/2}$ peak at 84.0 eV. The binding energy of the reported spectra is referenced to the C1s peak of saturated (sp^3) hydrocarbons at 285 eV. Spectra were processed using MultiPak software.

Fourier Transform Infrared (FTIR) spectra were measured using a Nicolet iS50 spectrometer (Thermo Fisher, Waltham, MA, USA) with an attenuated total reflectance (ATR) assessory. The FTIR ATR spectra were collected in the 4000 to 400 cm^{-1} wavenumber range, accumulating 128 scans.

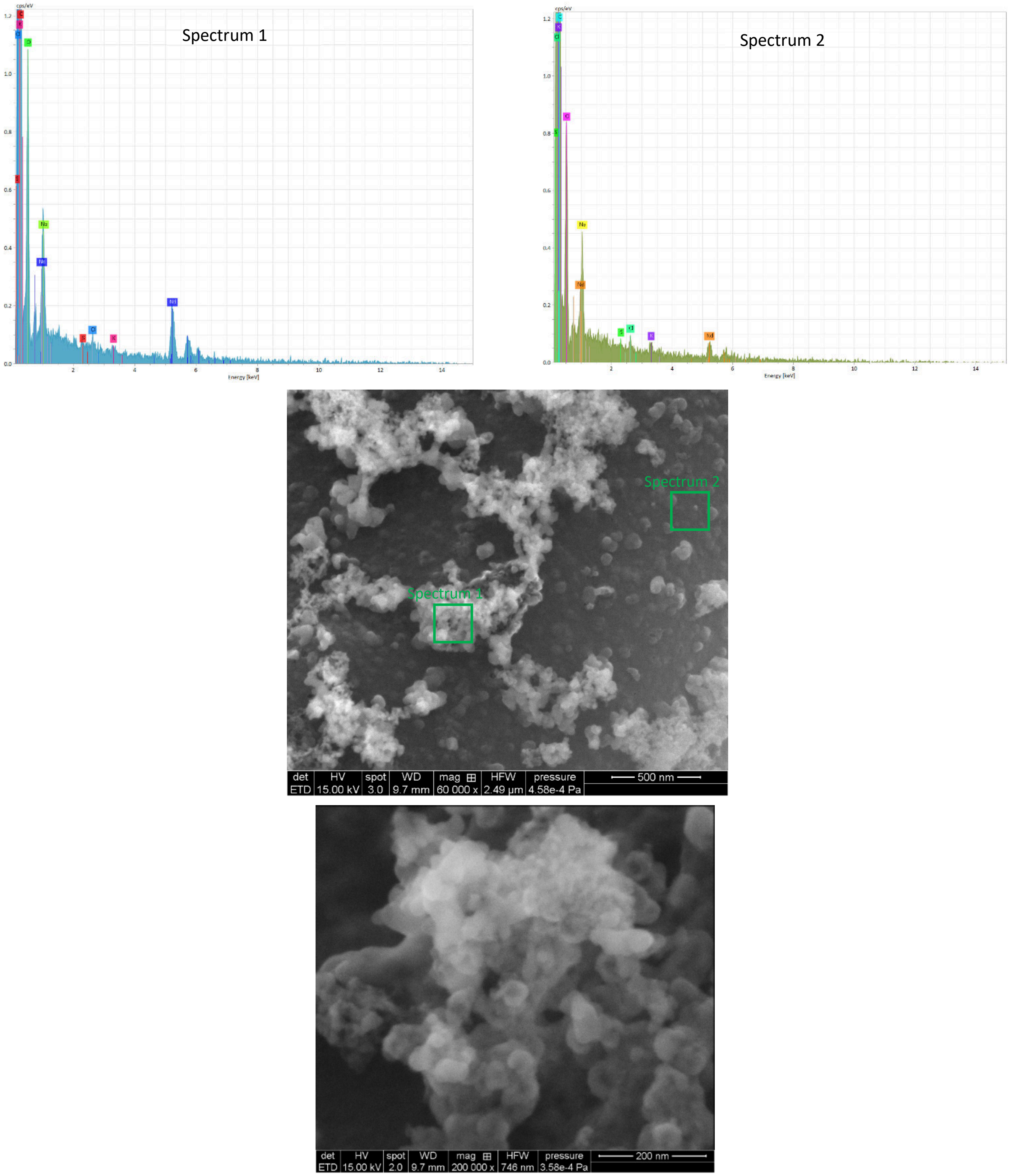

Figure S1. SEM/EDS of the Nd precipitate formed on a RVC cathode in SEP at −0.30 V in a 10 mg/L Nd solution in 0.01 M NaCl, initial pH 6, in a 120 mL cell.

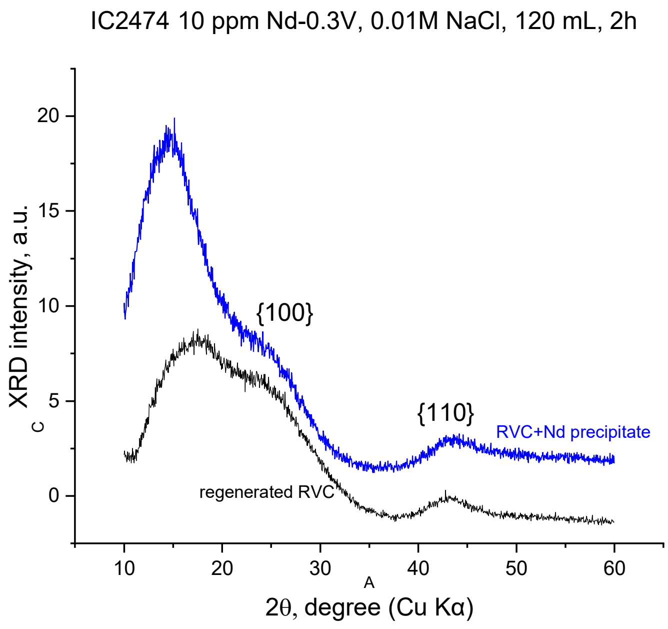

Figure S2 XRD of a regenerated RVC cathode as such and after SEP at –0.30 V in a 10 mg/L Nd solution in 0.01 M NaCl, initial pH 6, in a 120 mL cell. Its SEM images are shown in **Figure S1**.

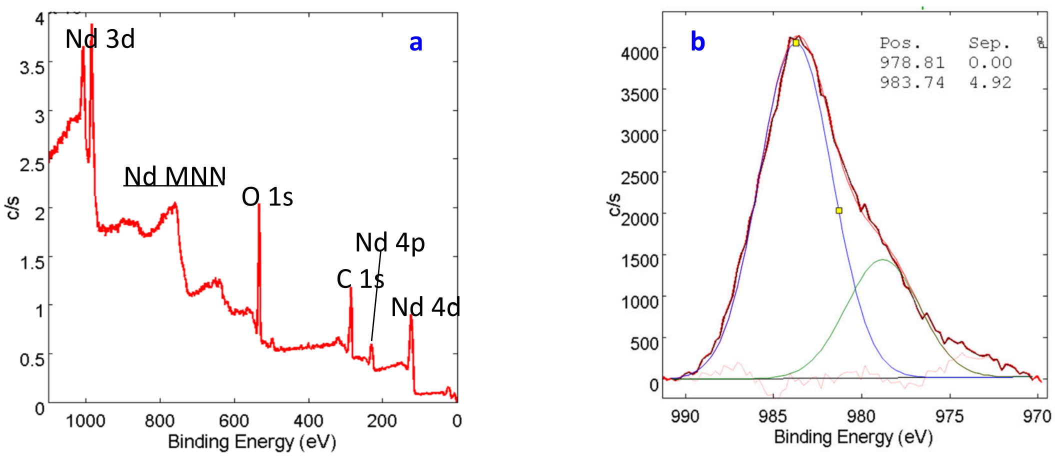

Figure S3 (continued)

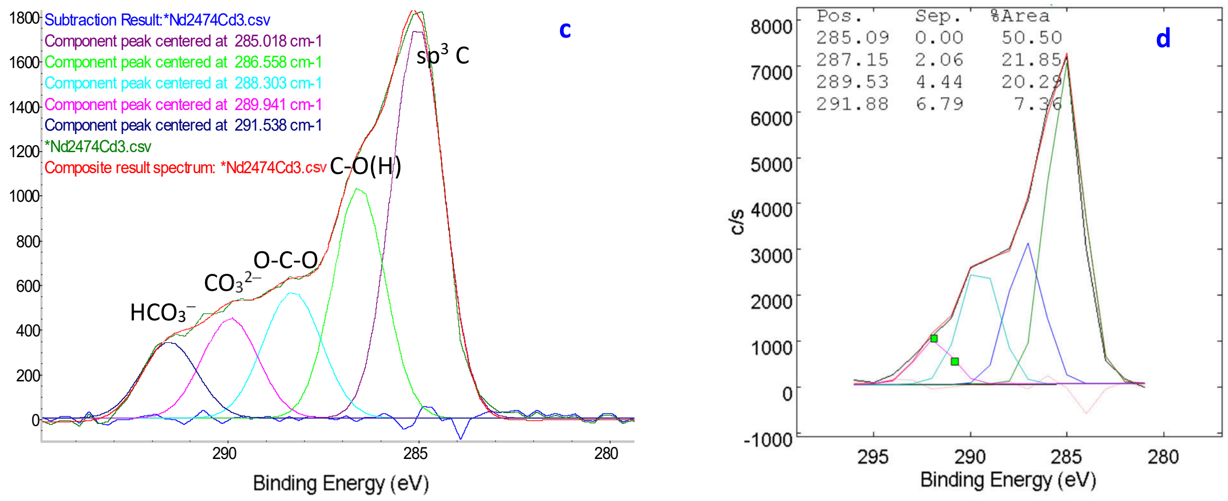

Figure S3 XPS spectra of the Nd precipitate formed on a RVC cathode in SEP at –0.30 V in 10 mg/L Nd solution in 0.01 M NaCl, initial pH 6, in a 120 mL cell: (**a**) survey spectrum, (**b**) regional Nd $3d_{5/2}$ spectrum, (c) regional C 1s. Its SEM and XRD are shown in Figure S1 and Figure S2, respectively.

Curve-fitting of the regional C 1s peak of the Nd precipitate formed on a RVC cathode in SEP at –0.30 V in **Figure S3c** resolves components at 285.0 eV (41%), 286.6 eV (24%), 288.3 eV (15%), 289.9 eV (11%) and 291.5 eV (8.5%). The first three peaks are assigned to sp^3 carbon, C-O(H) and O-C-O of organic contamination, respectively. The peak at 289.9 eV is assigned to carbonate given that anhydrous $La_2(CO_3)_3$ and $Ce_2(CO_3)_3$ are characterized by C 1s peaks at 289.8 eV and 289.7 eV, respectively (**xpsdatabase.net**). The peak at 291.5 eV can be assigned to bicarbonate ions, given that C 1s of $NaHCO_3$ is by 0.6 eV higher than that of Na_2CO_3.[1] Thus, the XPS spectra are consistent with the formation of Nd carbonate.

We noticed that the Nd carbonate is unstable under XPS conditions as its C 1s spectrum measured in the fast survey mode (**Figure S3d**) is different from the regional spectrum (**Figure S3c**). Specifically, the relative concentration of carbonate+bicarbonate carbon in the former is higher by ~50%, suggesting that the Nd (bi)carbonate is partially converted to Nd_2O_3. The ratio of carbonate+bicarbonate carbon to Nd using the C 1s spectrum measured in the fast survey mode (**Figure S3d**) **is 1.8**, which suggests the Nd phase is normal carbonate $Nd_2(CO_3)_3$ rather than basic carbonate.

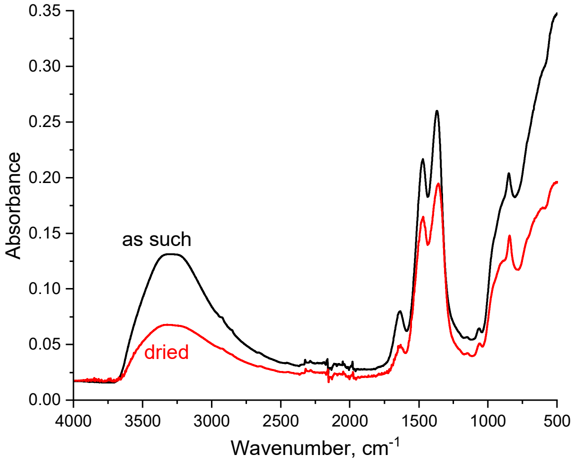

Figure S4. FTIR ATR spectra of the Nd precipitate obtained by titrating a 10 mg/L Nd solution in 0.01 M NaCl with 0.1 M NaOH up to pH ~9. The spectrum labeled 'as such' was obtained on a wet precipitate immediately after separating it from the solution using a syringe filter. The spectrum labeled 'dried' was obtained on the same sample after 15 min. These spectra are similar to those reported for amorphous normal Nd carbonate.[2,3] The band assignment is provided in Refs.[2,3]

Speciation Modelling using Visual Minteq 3.1 (https://vminteq.com/download/)

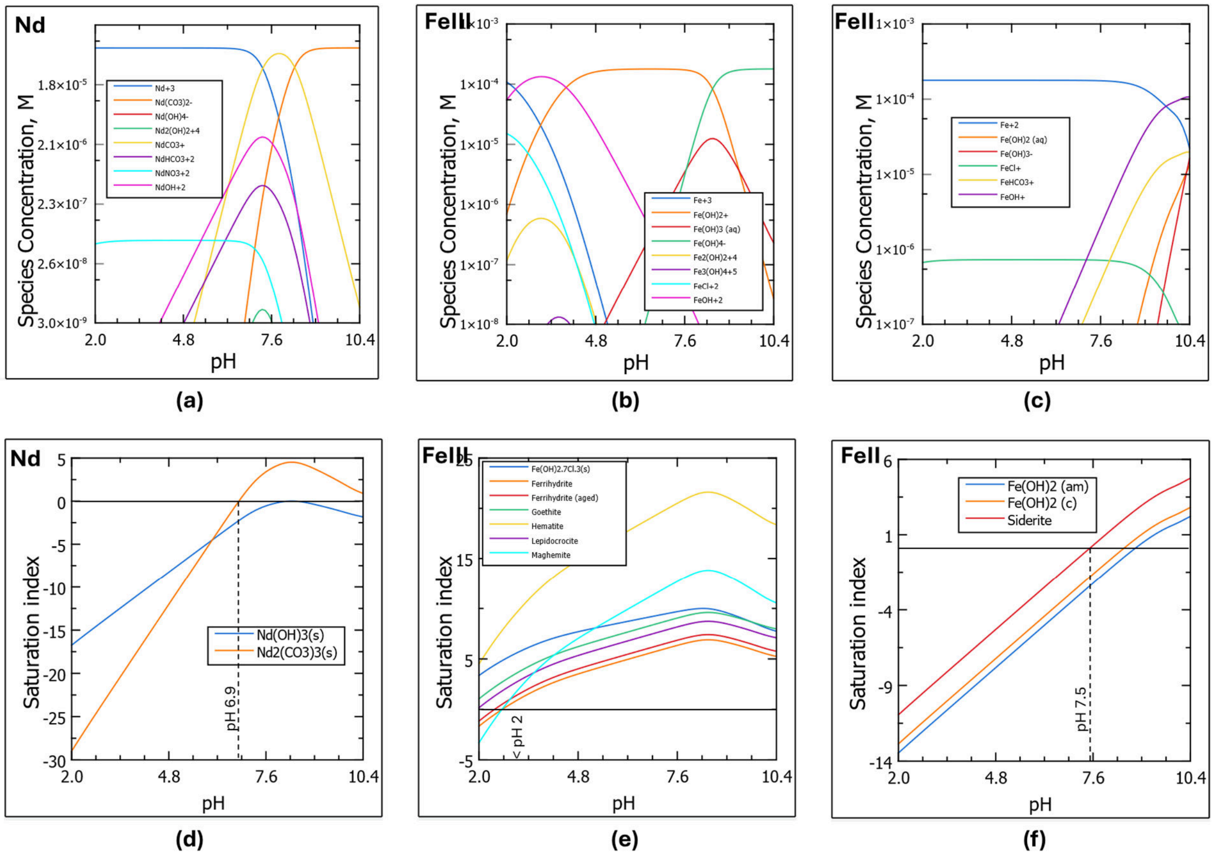

Figure S5 (continued)

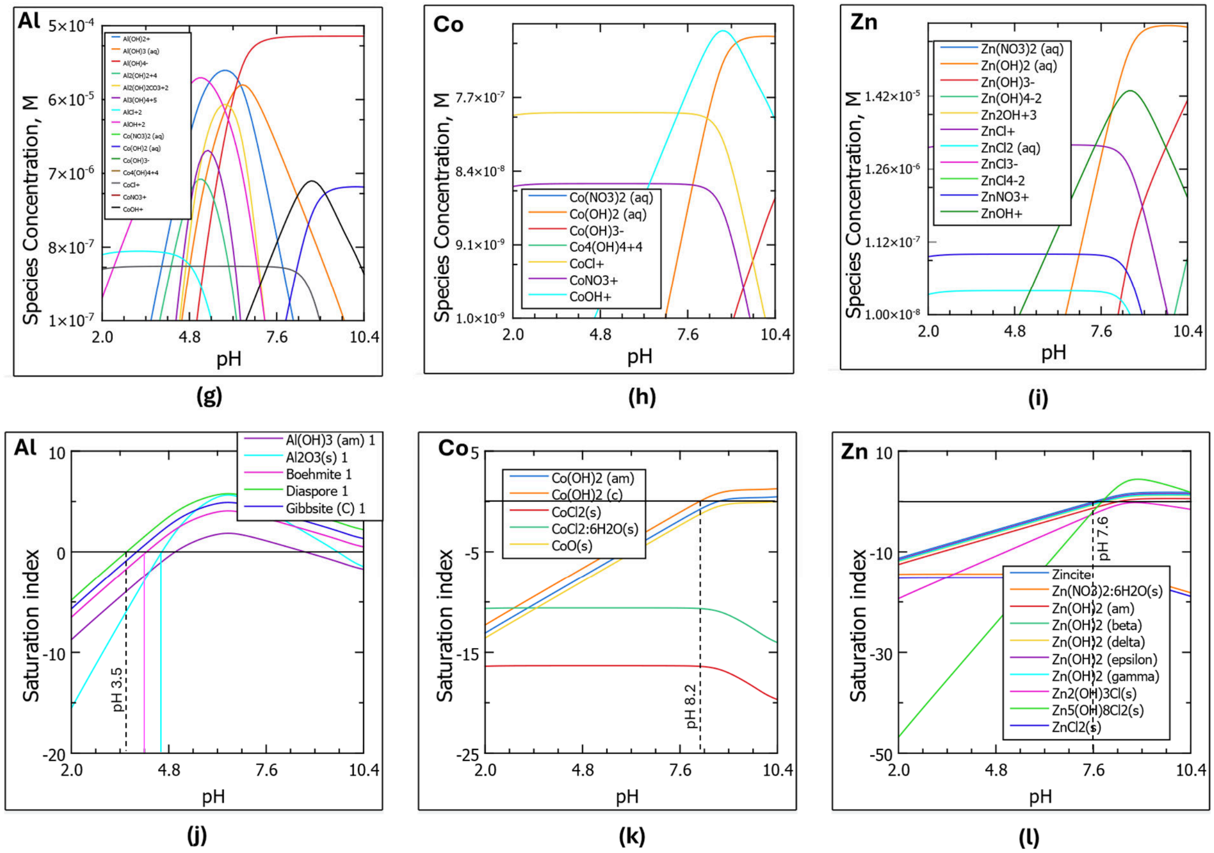

Figure S5 Speciation and saturation index of (a,d) Nd(III), (b,e) Fe(III), (c,f) Fe(II), (g,j) Al(III), (h,k) Co(II), and (i,l) Zn(II) at 10 mg/L in 0.01 M NaCl. These plots were generated using Visual Minteq 3.1 at a fixed partial CO_2 pressure of 0.0039 atm. The cations were added as nitrate salts corresponding to 0.0693 mM $Nd(NO_3)_3$, 0.179 mM $Fe(NO_3)_3$, 0.179mM $Fe(NO_3)_2$, 0.37 mM $Al(NO_3)_3$, 0.167 mM $Co(NO_3)_2$, and 0.153 mM $Zn(NO_3)_2$.

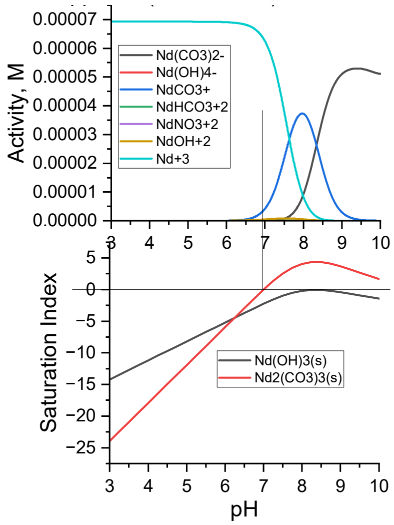

Figure S6 Speciation modelling for Nd in a 10 mg/L solution of Nd in 0.1 M NaCl. These plots were generated using Visual Minteq 3.1 at a fixed partial CO_2 pressure of 0.0039 atm. The cations were added as nitrate salts corresponding to 0.0693 mM $Nd(NO_3)_3$.

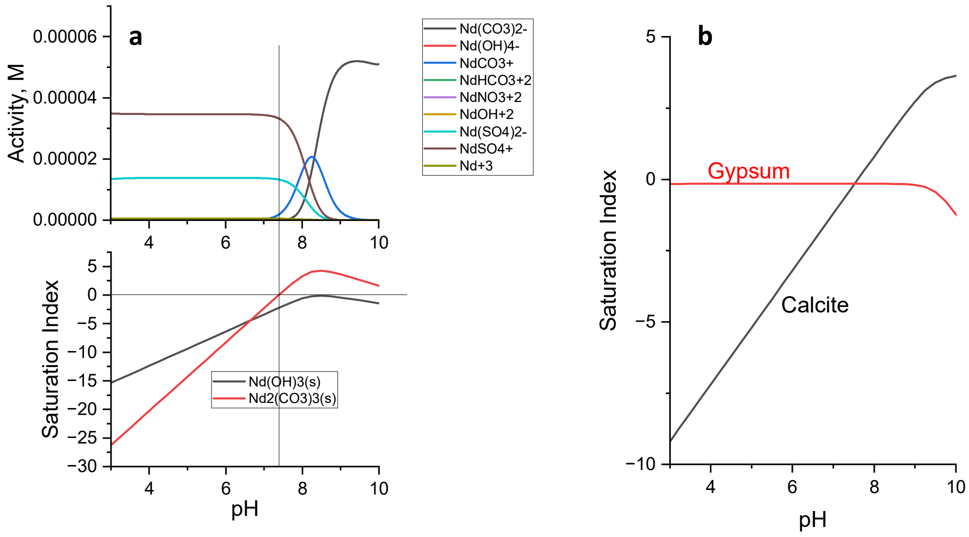

Figure S7. (**a**) Speciation modelling for a solution of 10 mg/L Nd in 0.05 Na_2SO_4. (**b**) Saturation index of gypsum and calcite in a solution of 10 mg/L Nd and 1 g/L Ca in 0.01 M NaCl. These plots were generated using Visual Minteq 3.1 at a fixed partial CO_2 pressure of 0.0039 atm. Ca is added as $CaCl_2$.

Chronoamperometric dependences

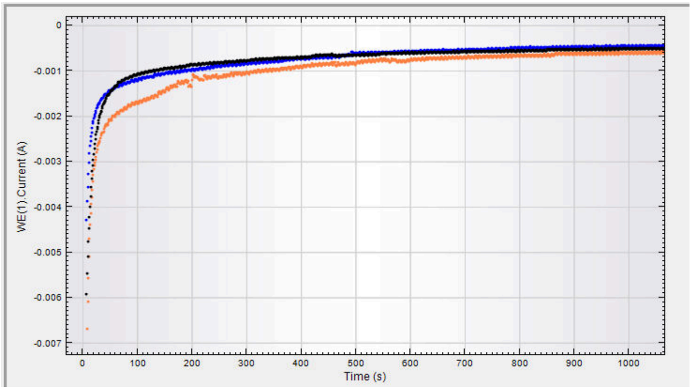

Figure S8. The effect of the background salt on the cathodic current at –0.30 V in a 10 mg/L Nd solution in 0.01 M (orange) Na_2SO_4, (blue) $NaNO_3$, and (black) NaCl at initial pH 7 during the tests shown in **Figure 2** of the main text.

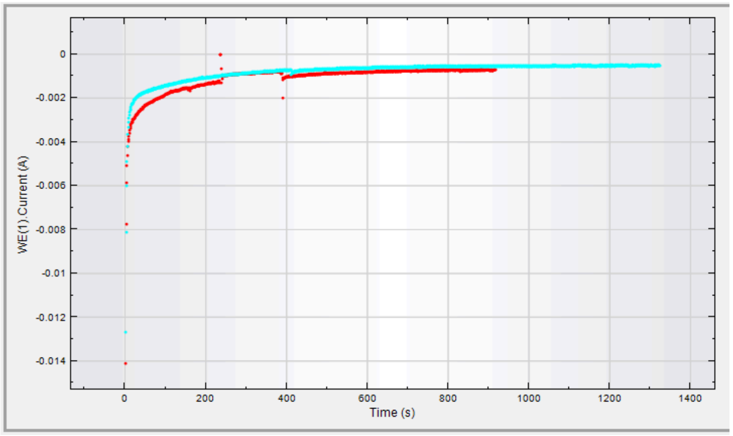

Figure S9. Effect of sulfate concentration on the cathodic current at –0.30 V in a 10 mg/L Nd solution in (red) 0.01 and (cyan) 0.0345 M Na_2SO_4 at initial pH 7.

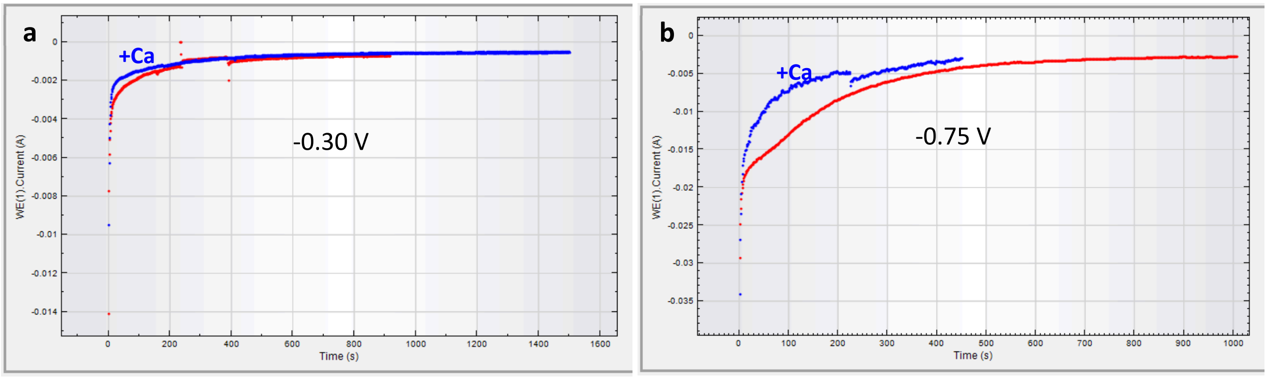

Figure S10. The inhibiting effect of 1 g/L Ca on the cathodic current in a 10 mg/L Nd solution in 0.01 M Na_2SO_4 at (a) –0.30 V and (b) –0.75 V. The test results are shown in **Figure 4a,b** of the main text.

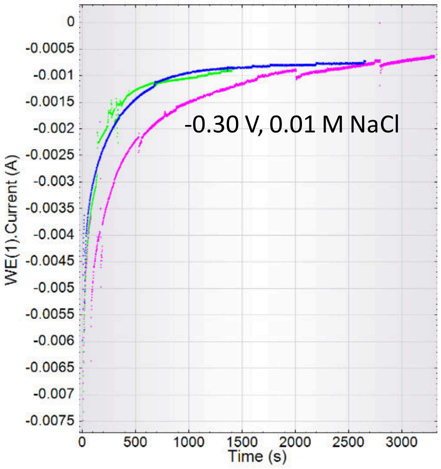

Figure S11. Cathodic current at –0.30 V in a solution of (blue) Nd + Al, (green) Nd+Fe(II), and (magenta) Nd+Fe(II)+Al+Zn, 10 mg/L each, in 0.01 M NaCl, pH 4.5. The test results are shown in **Figure 5** and **Figure 6a** of the main text.

References

(1) Shchukarev, A.; Korolkov, D. XPS Study of group IA carbonates. *Open Chemistry* **2004**, *2* (2), 347-362. DOI: doi:10.2478/BF02475578 (acccessed 2024-09-17).

(2) Cui, Z.; Guo, J.; Wang, D.; Cao, J.; Wang, Z. Stability of amorphous neodymium carbonate and morphology control of neodymium carbonate in non-hydrothermal synthesis. *J. Cryst. Growth* **2022**, *579*, 126460. DOI: https://doi.org/10.1016/j.jcrysgro.2021.126460.

(3) Vallina, B.; Rodriguez-Blanco, J. D.; Brown, A. P.; Blanco, J. A.; Benning, L. G. The role of amorphous precursors in the crystallization of La and Nd carbonates. *Nanoscale* **2015**, *7* (28), 12166-12179, 10.1039/C5NR01497B. DOI: 10.1039/C5NR01497B.
